# Assessment of Functional Activities in Individuals with Parkinson’s Disease Using a Simple and Reliable Smartphone-Based Procedure

**DOI:** 10.3390/ijerph17114123

**Published:** 2020-06-09

**Authors:** Pilar Serra-Añó, José Francisco Pedrero-Sánchez, Marta Inglés, Marta Aguilar-Rodríguez, Ismael Vargas-Villanueva, Juan López-Pascual

**Affiliations:** 1 UBIC, Departament de Fisioteràpia de la Universitat de València, 46010 València, Spain; pilar.serra@uv.es (P.S.-A.); marta.aguilar@uv.es (M.A.-R.); ismaelvargasvillanueva@gmail.com (I.V.-V.); 2 Instituto de Biomecánica de Valencia, Universitat Politècnica de València, 46021 València, Spain; jose.pedrero@ibv.org (J.F.P.-S.); juan.lopez@ibv.org (J.L.-P.); 3 Freshage Research Group, Department of Physiotherapy, Universitat de València, Centro de Investigación Biomédica en Red Fragilidad y Envejecimiento Saludable (CIBERFES-ISCIII), Fundación Investigación del Hospital Clínico Universitario de Valencia (INCLIVA), 46010 València, Spain

**Keywords:** Parkinson’s disease, smartphone, inertial measurement unit, functional mobility, gait, postural control

## Abstract

Parkinson’s disease (PD) is a progressive neurodegenerative disorder leading to functional impairment. In order to monitor the progression of the disease and to implement individualized therapeutic approaches, functional assessments are paramount. The aim of this study was to determine the impact of PD on balance, gait, turn-to-sit and sit-to-stand by means of a single short-duration reliable test using a single inertial measurement unit embedded in a smartphone device. Study participants included 29 individuals with mild-to moderate PD (PG) and 31 age-matched healthy counterparts (CG). Functional assessment with FallSkip^®^ included postural control (i.e., Medial-Lateral (ML) and Anterior-Posterior (AP) displacements), gait (Vertical (V) and Medial-Lateral (ML) ranges), turn-to-sit (time) and sit-to-stand (power) tests, total time and gait reaction time. Our results disclosed a reliable procedure (intra-class correlation coefficient (ICC) = 0.58–0.92). PG displayed significantly larger ML and AP displacements during the postural test, a decrease in ML range while walking and a longer time needed to perform the turn-to-sit task than CG (*p* < 0.05). No differences between groups were found for V range, sit-to-stand test, total time and reaction time (*p* > 0.05). In conclusion, people with mild-to-moderate PD exhibit impaired postural control, altered gait strategy and slower turn-to-sit performance than age-matched healthy people.

## 1. Introduction

Parkinson’s disease (PD) is a chronic and neurodegenerative pathology mainly characterized by motor symptoms (i.e., bradykinesia, rigidity, tremor and postural instability), that may lead to functional limitations associated with adverse events, such as falls, fractures and brain injuries [1,2]. PD constitutes a frequent cause of morbidity, and its prevalence increases with age, thus affecting 1% of the population over 60 years [3]. Due to the progressive deterioration of the overall condition of PD patients, measuring the functional status is of great importance not only to better control the degenerative process but also to suitably design customized therapeutic exercise programs, adapted to the particular characteristics of each patient rather than relying on group programs, these being the most commonly implemented. Indeed, previous studies have concluded that individual treatment ensures greater benefits than group therapy among people with PD [4].

An extensive amount of bibliography has addressed the assessment of functional activities like gait, sitting or balance using sophisticated systems and devices, which has been extremely useful to characterize the functional status in PD. However, most healthcare professionals dealing with this type of patient have no access to such technologies due to the high costs in addition to the space requirements not being adapted to the clinical setting. For this reason, developing objective cost-effective easy-to-administer functional tests (i.e., short-duration and portable technology) to assess daily life activities among people with PD would be helpful as a clinical tool to control the functional progression in these individuals and to adapt the therapeutic exercise programs to their particular functional condition.

In line with this, previous studies have shown the potential of using portable inertial measurement sensors (IMUs) for the assessment of daily life activities in people with PD. This technology has demonstrated how PD affects motor control in gait; in both straight-line walking and turning [5]; in balance; or in certain daily life movements, such as turn-to-sit maneuvers [6] or standing up from a chair [7,8]. IMU measurement is usually managed by means of a tablet or a PC which usually requires a more complex setting [9]. However, previous studies have disclosed that IMU sensors integrated in smartphones are also reliable and valid for kinematic assessment [6,10], which could be very interesting for healthcare professionals, in order to simplify the testing procedure. Indeed, our group previously provided the reliability of a modified Timed up-and-go test (TUG) using IMUs embedded in smartphones [11]. We also assessed the ability to perform daily life activities in patients suffering from another neurological disease, specifically Alzheimer’s Disease, thus providing not only the required time to complete the tasks of the test but also variables with a clinically relevant meaning (i.e., center of mass (COM) sway, gait instability and efficiency, reaction time, etc.) [12]. A recent study used a similar approach in PD [13] and obtained differences between PD patients and elderly people in the performance of the Timed up-and-go (TUG) test. However, their procedure did not include the measurement of balance, a very important clinical outcome in the evaluation of PD functional status [14]. In order to monitor the progression of the disease in the clinical setting and to design an individualized functional treatment plan, a test including different representative daily life activities capable of characterizing multiple function at a glance could be of interest. So far, to the best of our knowledge, there is no previous studies in which several functional activities, besides gait and balance, are assessed in a single test and which uses only a sensor embedded in a device in which the data is processed [15]. This approach could simplify the use of an objective assessment for the progression monitoring in a clinical context.

This study aimed to determine the ability to perform functional activities, such as balance, gait, turn-to-sit and sit-to-stand in patients with PD by means of a single short-duration test using a smartphone. Further, the reliability of such smartphone-based procedure in this population was studied.

## 2. Materials and Methods

### 2.1. Participants

Twenty-nine patients diagnosed with PD—according to the United Kingdom Bank Criteria [16]—were recruited for four months from outpatient clinics of several hospitals and from Parkinson’s associations, making up the PD group (PG). The inclusion criteria were diagnosis of mild to moderate Parkinson’s disease (Hoehn and Yahr stages 2 and 3 [17]); optimized and stable pharmacological therapy for at least 1 month before enrolment; and no cognitive impairment, as assessed by the Folstein Mini Mental State Exam (score ≥ 24) [18]. Further, volunteers were excluded if they presented other neurological or orthopedic impairments limiting independent gait and sitting down or getting up from a chair, Deep Brain Stimulation or Duodopa treatment. All patients were assessed in the “on” state one hour after taking medication.

During testing, there should be no dyskinesia, freezing or other types of involuntary movements. Thirty-one participants without neurological or motor disorders, matched for age and Body Mass Index (BMI), also participated in the study, making up the control group (CG).

The study was approved by the Ethical Committee of the University of Valencia (Nr. 1517239006520). All participants gave written informed consent. All the procedures were performed in accordance with the principles of the World Medical Association’s Declaration of Helsinki.

### 2.2. Sample Size Calculation

A priori sample size estimation was calculated using G*Power (Version 3.1.9.2; Kiel University, Germany) to determine the required number of volunteers. Alpha and beta errors were set at 0.05 and 0.20, respectively, and data from previous literature [19] which established a minimum detectable change in a similar test (i.e., Timed up and go) (4.85 s), were taken as the effect size. With this computation, a sample of 10 participants per group was required. Nevertheless, we included 29 and 31 participants in the PG and CG, respectively, since the recruitment period allowed us to obtain more volunteers than we required.

### 2.3. Reliability of the Assessment Protocol

Before the main study was conducted, a test–retest reliability assessment of the procedure was carried out with 20 volunteers taking part in the main study. They were assessed twice, with a 15-min interval, by two different raters to ensure consistency of the protocol performance. Both applied the whole protocol in the same theoretical way (i.e., instructions to the patients, familiarization, instrumentation and testing). Rater 1 was not present during the assessment performed by rater 2 and vice versa. Protocol details are explained below.

### 2.4. Smartphone-Based Functional Assessment

Participants were assessed in a single session. They were instructed to wear comfortable clothing and their usual walking shoes, to avoid vigorous exercise the day before the tests and to bring any necessary visual or auditory aids for the assessment.

Functional assessment was performed using the FallSkip^®^ system (Biomechanical Institute of Valencia, València, Spain), which is a software running on an Android smartphone (Xiaomi Redmi 4 x Model MAG138) [11]. The data were acquired via the device sensor, specifically, High Performance 6-Axis MEMS MotionTracking™ composed of 3-axis gyroscope (gyro); 3-axis accelerometer (acc); and a Digital Motion Processor™ (TDK-ICM-20689) at 100 Hz, which provides high robustness by supporting 20,000 g shock reliability, according to the manufacturer information A custom specific software was developed in Python to calculate the variables from the raw sensor data. First of all, the height and weight of the participants were measured and subsequently used to calculate the clinical dependent variables. The device was then attached horizontally with an elastic belt just below the posterior superior iliac crests.

**Figure 1:**
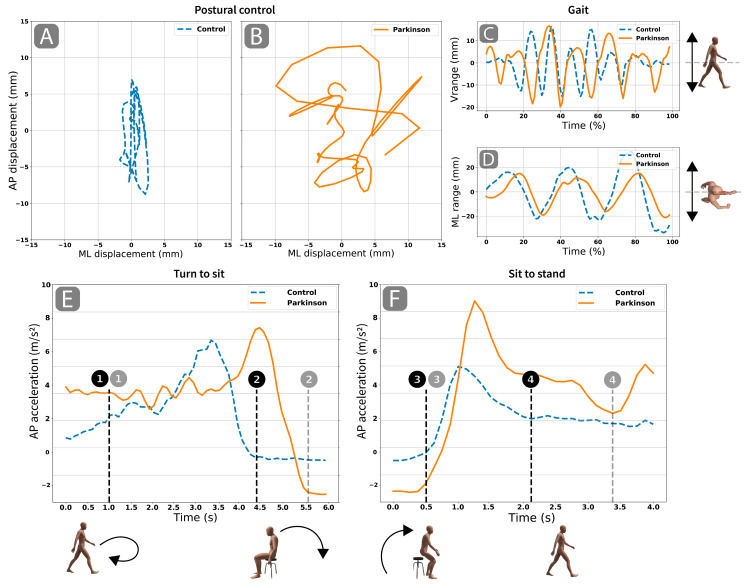
Graphical presentation of the postural control, gait, turn-to-sit and sit-to-stand subtests. An example of one participant per group for each variable is represented. The blue dotted line is data from the control group; the solid orange line is data from the Parkinson’s group. (**A**,**B**): anterior-posterior (AP) and medial-lateral (ML) displacement of the center of mass, respectively, in the balance subtest. (**C**): vertical displacement of the center of mass in the gait subtask that is also represented by the sagittal view of the dummy. (**D**): medial-lateral displacement of the center of mass in the gait subtest depicted by a zenithal view of the dummy. (**E,F**): accelerometer signals in the vertical axis for the turn-to-sit and sit-to-stand tasks, respectively. Benchmarks of the turn-to-sit and sit-to-stand tasks which are also illustrated by the four dummies: **1**, start of the turn-and-sit subtest of both participants; **2**, end of sitting; **3**, start of the sit-to-stand subtest; **4**, end of the sit-to-stand and start of walking back to the starting point.

With this device, a functional test procedure was conducted (Figure 1). This procedure included postural control (Figure 1A,B), locomotion (Figure 1C,D), turn-to-sit (Figure 1E) and sit-to-stand subtests (Figure 1F) [12]. Firstly, the participants should remain in a bipedal stance with their arms hanging relaxed alongside their body for 30 s. An acoustic signal then sounded, and the participant should have immediately started walking a 3-m stretch as fast as was safely possible. When the distance was covered, they should stop for 3 s and then turn around and sit down on a chair. They should remain seated for 3 s before getting up and returning to the starting position. 

The rater stood behind the participants during the whole test to assist participants, if required. Furthermore, several trials were conducted before the test started to allow the participants to familiarize themselves with the test.

### 2.5. Smartphone Data Analysis and Outcomes

All raw sensor data were processed according to the procedures explained in our previous study [12]. First of all, time events were manually identified to split up the recorded data into the tasks under study [12]. Based on the raw data from the sensors, postural control, gait and functionality variables were calculated. Figure 1A–F shows a representation of the sensors data in each subtest. To better illustrate the performance of each group, we selected two individuals for each variable (one from the CG and one from the PG). The individuals selected in each case corresponded to the 50th percentile of the variable.

A panel plot was built as an example of the data recorded during each subtest (Figure 1). For 160 each panel (Figure 1A–F), one individual from the CG and one from the PG were plotted. First, we selected the representative individuals identifying the case corresponding to the 50th percentile of each group in each discrete variable. Further, we plotted the piece of data used to compute each discrete variable.

Two variables were calculated for the postural control subtest: (1) Medial-lateral displacement (MLDisp): 90th percentile of the medial-lateral excursion of the COM, measured in mm and (2) Anterior-posterior displacement (APDisp): 90th percentile of the anterior-posterior excursion of COM. Both variables are calculated by double integration of the acc signal [20] and an inverted pendulum model [21]. Both are common variables used in the assessment of the postural steadiness in terms of COM displacement [22].

For the gait subtest, two variables were measured: 1) Vertical range (Vrange): vertical COM movement, measured in mm (Figure 1C and associated lateral view of the dummy) and 2) Medial-lateral range (MLrange): horizontal COM movement, measured in mm. (Figure 1D and associated zenithal view of the dummy). Both were calculated by double integration of the acc signal [23]. Vrange is representative of the energy cost [23,24], while MLrange, in addition to the energy cost, refers to the dynamic stability while walking. 

Furthermore, turning around and sitting down, and getting up from a chair were also studied with two variables: (1) Turn-to-sit time (TTurnSit): estimated time in seconds required to turn around and sit in a chair (Figure 1E and associated dummies) and (2) Sit-to-stand power (PStand): estimated mean power, measured in watts, generated by getting up from the chair (Figure 1E and associated dummies). Both variables were estimated by the participant’s COM trajectory, and weight and height during the movements [25]. These are complex motor daily life activities that require cognitive planning and coordination of the neuromuscular systems to regulate COM displacement [26]. 

**Figure 2:**
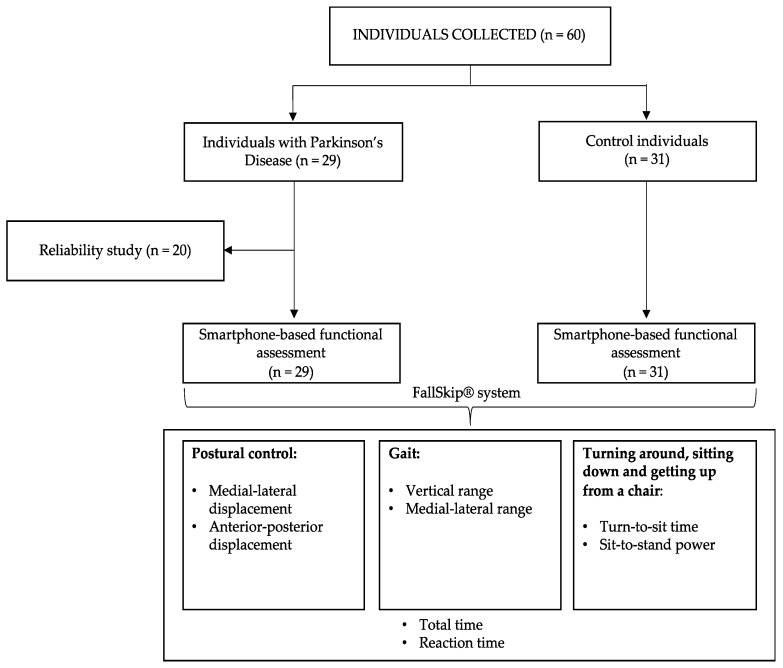
Flowchart of the experimental design and assessment procedures.

Finally, we computed the time required to complete all the subtests (total time), as measured in seconds, and the time elapsed from the acoustic signal to gait initiation (reaction time). Speed is the most common variable used when describing gait [27], and reaction time has been proved useful in predicting freeze in gait initiation [28]. Figure 2 summarizes the experimental design and assessment procedures of the study.

### 2.6. Statistics

Statistical analysis was performed using SPSS software Version 24 (SPSS Inc., Chicago, IL, USA). Standard statistical methods were used to obtain the mean as a measure of central tendency and the standard deviation (SD) as a measure of dispersion. For comparison of the functional outcomes between CG and PG, an independent t-test was conducted. When homoscedasticity was violated (i.e., in the APDispl and TTurnSit variables), the Welch–Satterthwaite approximation was used. Nevertheless, the use of this approximation did not modify the results (see Supplementary Material: Table S1).

The reliability of the functional procedure was determined using a repeated-measures analysis of variance (ANOVA) to calculate the (2,1) intra-class correlation coefficient (ICC) [29]. A *p*-value of 0.05 was accepted as the level of significance.

## 3. Results

### 3.1. Participants

The PG included 29 individuals with a mean (SD) age of 68.9 (8.98) years. The CG, with 31 individuals, showed a mean (SD) age of 67.23 (8.16) years. There were no significant differences (*p* > 0.05) between groups in weight (74.9 (12.71) vs 76.71 (13.11) kg) and height (1.68 (0.08) vs 1.65 (0.07) m).

### 3.2. Reliability Study

Twenty people with PD in the main study volunteered to participate in the reliability study. As noted in Table 1, reliability was good for the postural control variables (i.e., MLDispl and APDispl); excellent for the gait (i.e., VRange and MLRange), PStand and TTurnSit variables; and fair for the reaction time variable [30,31].

### 3.3. Functional Assessment Comparison

Table 2 shows the descriptive and inferential analysis comparing the functional outcomes between groups (i.e., CG and PG). As shown, there were significant differences between groups in postural control variables, since MLDisp and APDisp based on the COM were significantly larger in PG than in healthy counterparts. The average time to perform the assessment was 3:27 (1:12) min. Specific results for the Levene test and the Independent t-test can be consulted in the Supplementary Material.

## 4. Discussion

This study reports a single quick and easy-to-use test assessing a number of daily life activities that are capable of identifying functional differences between a PD population and their healthy counterparts. Indeed, people with PD showed a larger COM displacement when they remained in a static bipedal stance, with even larger values in medial-lateral and anterior-posterior displacement. PD subjects further exhibited altered gait, as indicated by a lower MLRange, although no changes in VRange were found. Additionally, they required a significantly longer time to turn and sit in a chair although the total time required to perform the test was similar in both groups, as was the reaction time.

PD is a progressive chronic disease for which no cure has yet been found, but progression of its symptoms can be alleviated or even slowed down by means of suitable pharmacological and non-pharmacological interventions, such as physiotherapy and physical exercise [32]. Indeed, several systematic reviews and meta-analyses have reported how these non-pharmacological approaches are able to improve a wide range of motor symptoms in PD, including strength, gait and balance [33,34,35]. Thus, monitoring the clinical functional outcomes of such interventions becomes a paramount challenge for health professionals in order to better adapt the treatment to the particular characteristics of each patient. Moreover, long-term monitoring ought to be performed throughout the course of the disease to detect the most relevant features and to adapt the therapeutic plans to the progression [36]. Currently, many tools have been described for the assessment of functional status in people with PD. Some are regularly used in a clinical context, such as scales and questionnaires. However, some scales commonly used to address functional status, such as the Berg Balance Scale for instance, may offer limited utility in middle-stage Parkinson’s Disease (Hoen and Yahr Stages 2 to 3) due to ceiling effects [37] or their long duration (15–20 min). Conversely, sophisticated and precise systems, such as video-photogrammetry or force plates, are extremely useful to precisely characterize the disease but are not easy to implement in the clinical setting due to their high costs and space requirements. Besides, the patients are forced to visit a laboratory, which causes a real-life assessment to be difficult to reproduce. Therefore, the development of an objective wearable and an easy-to-use device, as employed in our study, would be of interest to assess functional status (e.g., postural control, gait, sit-to-stand and turn-to-sit) in people with PD even in real situations [38].

The protocol used in this study has been proven to be reliable in a previous study conducted by our group in people with Alzheimer’s [11]. Likewise, in the current study, a good to excellent ICC was observed in all variables but one, namely reaction time, which was fair.

This protocol addressed different daily life activities. One of them, postural instability, has been proposed as one of the hallmarks of the disease [2] and a major factor determining quality of life and morbidity in people with PD [14]. However, clinicians and patients have traditionally focused mainly on gait impairments. Postural control involves active brain processes that integrate information from all levels of the musculoskeletal and nervous systems, when moving (dynamic balance) and when standing motionless (static balance) [38].

Our results disclosed that people with PD displayed higher values of COM displacement, both in the medial-lateral and the anterior-posterior directions. This is in line with previous studies that have determined greater displacements of the center of pressure [39,40] in people with PD using force plates. This postural alteration observed in people with PD may be associated with basal ganglia dysfunction, a key pathologic structure in PD, involved in balance control, automated postural responses and gait, via the thalamic-cortical-spinal loops and via the brainstem pedunculopontine nucleus and the reticulospinal system [41]. Its alteration would thus justify the alteration of postural control and accordingly increased COM displacement. This is of particular interest because the increased sway and specifically the mediolateral postural sway in patients with PD at Hoen and Yahr Stages 1 to 3 have been shown to be a useful clinical observation to identify postural instability and increased risk of falls [42].

When gait analysis was performed, two variables were assessed: VRange and MLRange. VRange was determined to explore the metabolic cost during gait [43]. Based on the inverted pendulum theory [44], whereby the stance leg acts as an inverted pendulum during gait, a certain amount of vertical lift of the center of mass is needed which would reflect the exchange between potential and kinetic energy during each stride. Our results disclosed no significant differences between groups. The average value of VRange of the PG is similar to that obtained in a previous study conducted by our group [12]. According to this theory, we cannot affirm that the metabolic cost during gait differs between groups. However, this study pooled two different stages of PD. Further studies including more people at different stages of the disease could provide more information about this feature of gait.

Regarding the other gait-related variable, MLRange, a statistically significant decrease of 29.79% was found in the PG while walking. MLRange while walking reflects the amount of side-to-side frontal plane motion during each gait cycle and provides information about the ability to control body movement during gait tasks [45]. Furthermore, this lateral movement and, thus, lateral stabilization are required when walking [46]. In traumatic brain injury patients, such as people who have suffered a concussion, this lateral displacement has been proved enlarged, which reflects disruption in their ability to control their COM displacements [47]. The decrease obtained in our results might indicate that people with PD who undergo a progressive impairment of gait develop compensatory strategies to avoid falls, like reducing the natural medial-lateral oscillation when walking. Indeed, previous studies have demonstrated scarce dissociation between head, trunk and lower limbs [48,49]; therefore, the natural movement of the pelvis during gait in the frontal plane could be jeopardized. This result suggests that therapeutic exercises focused on the dissociation of the shoulder and pelvic girdle would be of interest in this population. Future studies accounting for more advanced stages of the disease might clarify whether this strategy is maintained as the disease progresses or if disruption occurs in the control of medial-lateral transfers of center of mass. This would allow clinicians to perform an adequate readjustment of functional treatments and therapeutic exercises.

The sit-to-stand task is an essential activity that requires a proper neuromuscular system coordination to control COM displacement and postural alignment. There is controversy about the influence of PD on the performance of the sit-to-stand task. A previous study reported overall slower transfers from sit-to-stand but a similar peak velocity to that obtained from their healthy counter-parts [50]. Another study reported a similar duration of this daily task in the PD population as in healthy individuals but a greater preparatory hip flexion displacement, forward center of mass displacement and reduced knee extensor moments [51]. Nevertheless, Mak et al. reported a similar kinematic pattern between people with PD and their healthy counterparts [52].

Our study reports that people with PD present a power reduction of 24.95%, but there were no significant differences between groups. These results are in line with those reported by Rojas et al., who also used a smartphone device and did not obtain significant differences between people with PD and healthy counterparts, neither in velocity nor in the time required to perform the task [8]. The fact that one of the test inclusion criteria is the ability to stand up without using your arms may introduce a clear floor effect in this variable. Further studies with larger samples may be of interest to explore whether the sit-to-stand power varies for the different Parkinson’s disease stages.

We also assessed sitting following turning around because it is more representative of daily life movements, since the body needs to be prepared at a suitable orientation in the chair before sitting. In our study, PG needed a longer time to perform the turn-to-sit task than that required by the CG. Our results are in line with those obtained in a recent study in which turning and sitting were studied independently, thus showing longer lapses of time to perform each of those tasks [13] However, our values are not entirely comparable because no previous study has analyzed the time required to perform both tasks together. Turning and sitting as a combined movement involves inter-limb coordination and modification of locomotor patterns and requires frontal lobe cognitive executive function and attention [26]. Planning-related neural activation and functional connectivity, however, have been proven to be reduced in people with PD [53]. This may explain why more time to perform the task is needed in people with PD, so monitoring this task throughout disease progression may provide valuable information on brain deterioration in PD.

With regard to the total time required to complete the whole test, no differences were noted between groups and both required approximately 15 s in all. There is controversy as to whether a similar test (i.e., TUG) is able to reliably differentiate people with PD and healthy counterparts [54,55]. In fact, a previous review concluded that TUG has limited sensitivity for predicting falls and should not be used alone to identify individuals at high risk of falls [56]. As discussed in the Introduction section, having more data beyond the required time to perform the test (i.e., biomechanical variables) might be useful to better characterize functional task biomechanical patterns and thus to better plan intervention approaches.

People with PD usually experience start hesitation, this is, a brief episode of freezing of gait in which step initiation is delayed, probably sustained by decoupling between the postural control and step and because of the inhibitory deficits that occur in this disease [57]. Interestingly, this feature has been previously associated with the risk of falls due to the fact that, during a delayed step onset, the COM continues moving forward [28]. Thus, we assessed reaction time between the acoustic sound and the start of gait and found similar results in people with PD and their age-matched counterparts, while none of our participants experienced freezing at any time during the test. This may be due to the particular characteristics of our sample including individuals at a mild to moderate stage of the disease, as freezing of gait has been related to advanced PD [58], so further studies including people with freeze episodes (i.e., advanced stages of the disease) could provide information on the utility of this variable in people with PD.

Having assessed all these functional activities in a single test using only one wearable sensor easily attached to the lower back supposes a qualitative leap in the clinical assessment of functional status in people with PD. Up to now, previous studies focused on specific functional assessments and most of them require several sensors paired to an external device.

Admittedly, this study had some limitations, including strict exclusion criteria and large intersubject variability since two stages of PD are included. On the contrary, the usefulness of the proposed procedure has not been determined in more advanced stages of PD. Further, we assessed people with PD in a medication “on” state. Therefore, our results cannot be extended beyond this condition. Besides, this study did not evaluate patients with active levodopa-induced dyskinesia and/or unpredictable motor fluctuations, which are common problems in the overall PD population. Another limitation is that we conducted a purposive sampling to the recruitment of the volunteers instead of a simple randomization method. Finally, we did not conduct a follow-up study in which the effect of the progression was studied. Instead, we assessed the effect of PD on functional variables in a single assessment, so the results could be influenced by the occasional variability of the patients. Further studies should test the value of this procedure in longitudinal studies. Despite these limitations, this study showed that patients with mild-to-moderate PD may have already developed impaired postural control, gait alterations and impaired ability to sit on a chair.

## 5. Conclusions

We have objectified by a simple smartphone-based procedure that people with mild- to moderate-stage PD display impaired postural control represented by larger COM displacements, an altered gait strategy in which the medial-lateral displacement is reduced and more time needed to conduct a turn-to-sit task than healthy age-matched people. Further, responding to the secondary goal of the study, the proposed procedure shows a high reliability so it can be used in people with PD.

## Figures and Tables

**Table 1:** Descriptive and reliability data from the test–retest study.

	Observer 1	Observer 2	ICC
Medial-lateral displacement (mm)	13.16 (9.33)	16.11 (12.57)	0.71
Anterior-posterior displacement (mm)	31.62 (13.26)	34.15 (20.77)	0.62
Vertical range (mm)	24.13 (8.00)	22.58 (7.28)	0.92
Medial-lateral range (mm)	25.63 (13.23)	25.28 (16.11)	0.89
Turn-to-sit time (s)	76.92 (25.66)	86.67 (30.43)	0.89
Sit-to-stand power (W)	4.98 (1.69)	4.42 (1.48)	0.82
Total time (s)	14.69 (3.13)	14.32 (3.33)	0.94
Reaction time (s)	1.08 (0.40)	0.93 (0.31)	0.58

Data are expressed as mean (SD); ICC: Intra-class correlation coefficient.

**Table 2:** Comparison of functional-related outcomes between people with Parkinson’s disease and healthy counterparts.

	CG (*n* = 31)	PG (*n* = 29)	*p*-Value	Cohens’ d
Medial-lateral displacement (mm)	8.44 (4.61)	11.38 (5.14) *	0.02	0.58
Anterior-posterior displacement (mm)	17.57 (6.34)	27.6 (13.15) *	<0.01	0.88
Vertical range (mm)	20.43 (4.5)	22.89 (7.32)	0.12	-
Medial-lateral range (mm)	39.67 (25.72)	27.85 (17.01) *	0.04	0.52
Turn-to-sit time (s)	4.17 (1.01)	5.21 (1.74) *	<0.01	0.70
Sit-to-stand power (W)	210.83 (67.11)	206.42 (66.06)	0.80	-
Total time (s)	15.15 (2.87)	15.2 (3.04)	0.95	-
Reaction time (s)	1.04 (0.36)	1.07 (0.39)	0.74	-

Data are expressed as mean (SD); CG, control group; PG, group of people with Parkinson’s disease; * significant differences between groups (*p* < 0.05); Cohen’s d size effect of the differences between groups (reported only when significant differences existed).

## Supplementary Materials

The following are available online at https://www.mdpi.com/1660-4601/17/11/4123/s1, Table S1: Specific results for the Levene test and the Independent t-test.
