# Supplementary material for: Assessment of Functional Activities in Individuals with Parkinson’s Disease Using a Simple and Reliable Smartphone-Based Procedure
Source: Int J Environ Res Public Health. 2020 Jun 9;17(11):4123. doi: 10.3390/ijerph17114123 (PMC7312659; doi:10.3390/ijerph17114123)
Supplement: Supplementary file 1 [file ijerph-17-04123-s001.pdf]

International Journal of Environmental Research and Public Health

MDPI

Table 1. Specific results for the Levene test and the Independent t-test.

**Variable**	Levene test (Assumption Met when *p* > 0.05)		Situation Regarding the Homoscedasticity	Independent t-test				
	F	*p*-value		t	Degrees of freedom	*p*-value	Mean difference	Standard error of the difference
**MLDispl**	0.156	0.694	Similar variance assumption	−2.329	58	0.023	−2.93152	1.25865
			No Similar variance assumption	−2.321	56.282	0.024	−2.93152	1.26324
APDispl	10.126	0.002	Similar variance assumption	−3.802	58	0.000	−10.03122	2.63820
			No Similar variance assumption	−3.723	39.733	0.001	−10.03122	2.69457
Vrange	2.557	0.115	Similar variance assumption	−1.584	58	0.119	−2.46679	1.55691
			No Similar variance assumption	−1.560	45.966	0.126	−2.46679	1.58089
MLrange	3.862	0.054	Similar variance assumption	2.084	58	0.042	11.81907	5.67070
			No Similar variance assumption	2.112	52.358	0.039	11.81907	5.59599
PStand	0.098	0.755	Similar variance assumption	0.256	58	0.799	4.40433	17.20681
			No Similar variance assumption	0.256	57.843	0.799	4.40433	17.19765
TTurnSit	5.392	0.024	Similar variance assumption	−2.869	58	0.006	−1.04506	0.36425
			No Similar variance assumption	−2.821	44.237	0.007	−1.04506	0.37046
Reaction time	0.147	0.703	Similar variance assumption	−0.333	58	0.740	−0.03191	0.09583
			No Similar variance assumption	−0.332	56.802	0.741	−0.03191	0.09608
Total time	0.000	0.992	Similar variance assumption	−0.060	58	0.953	−0.04558	0.76299
			No Similar variance assumption	−0.060	57.127	0.953	−0.04558	0.76443

Medial-lateral displacement (MLDisp). anterior-posterior displacement (APDisp). vertical range (Vrange). medial-lateral range (MLrange). Turn-to-sit time (TTurnSit). Sit-to-stand power (PStand).
